# Clinical Manifestations, Pathogenesis and Treatment of Hepatitis E Virus Infections

**DOI:** 10.3390/jcm9020331

**Published:** 2020-01-24

**Authors:** Sébastien Lhomme, Olivier Marion, Florence Abravanel, Jacques Izopet, Nassim Kamar

**Affiliations:** 1 Virology Laboratory, National Reference Center for Hepatitis E Virus, Toulouse Purpan University Hospital, 31300 Toulouse, France; abravanel.f@chu-toulouse.fr (F.A.); izopet.j@chu-toulouse.fr (J.I.); 2 INSERM UMR1043, Center for Pathophysiology of Toulouse Purpan, 31300 Toulouse, France; marion.o@chu-toulouse.fr; 3 Université Toulouse III Paul Sabatier, 31330 Toulouse, France; 4 Department of Nephrology and Organs Transplantation, Toulouse Rangueil University Hospital, 31400 Toulouse, France

**Keywords:** hepatitis E virus, acute hepatitis, chronic hepatitis, neurological disorders, renal injury, pregnancy

## Abstract

Hepatitis E virus (HEV) is the most common cause of acute viral hepatitis throughout the world. Most infections are acute but they can become chronic in immunocompromised patients, such as solid organ transplant patients, patients with hematologic malignancy undergoing chemotherapy and those with a human immunodeficiency virus (HIV) infection. Extra-hepatic manifestations, especially neurological and renal diseases, have also been described. To date, four main genotypes of HEV (HEV1-4) were described. HEV1 and HEV2 only infect humans, while HEV3 and HEV4 can infect both humans and animals, like pigs, wild boar, deer and rabbits. The real epidemiology of HEV has been underestimated because most infections are asymptomatic. This review focuses on the recent advances in our understanding of the pathophysiology of acute HEV infections, including severe hepatitis in patients with pre-existing liver disease and pregnant women. It also examines the mechanisms leading to chronic infection in immunocompromised patients and extra-hepatic manifestations. Acute infections are usually self-limiting and do not require antiviral treatment. Conversely, a chronic HEV infection can be cleared by decreasing the dose of immunosuppressive drugs or by treating with ribavirin for 3 months. Nevertheless, new drugs are needed for those cases in which ribavirin treatment fails.

## 1. Introduction

The hepatitis E virus (HEV) is the leading cause of acute viral hepatitis worldwide. HEV, which is mainly transmitted enterically, is responsible for outbreaks in developing countries and zoonotic cases in both developing and developed countries [1]. HEV genotypes 1 (HEV1) and 2 (HEV2) are mainly found in developing countries and are restricted to humans. Other genotypes, including HEV3 and HEV4, have been detected in both humans and animals, with pigs being the main reservoir. While most infections are asymptomatic, they can cause acute hepatitis, including severe forms in patients with pre-existing liver disease and in pregnant women living in developing countries. An HEV infection may also trigger extra-hepatic manifestations and can lead to chronic infections in immunocompromised patients. Its pathogenesis is still unclear but our knowledge has greatly improved in the past few years.

## 2. HEV Genome and Classification

**Figure 1:**
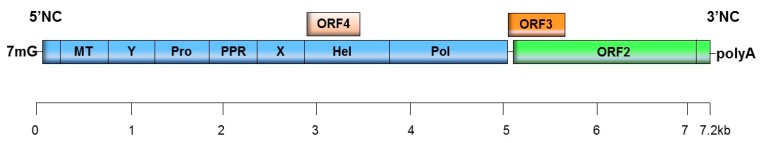
Hepatitis E virus (HEV) genome. Open reading frames (ORF1) (blue box) encodes nonstructural proteins. ORF4 has been found only in HEV1. 7 mG: 7-methylguanosine; Hel: helicase; MT: methyl transferase; polyA: polyadenylated tail; PPR: polyproline region; Pro: cysteine protease; RdRp: RNA polymerase; X: X domain; Y: Y domain.

HEV is a small virus with the positive-sense, single-stranded ~7.2 kb RNA genome that contains three open reading frames (ORF), ORF1, ORF2 and ORF3 (Figure 1). ORF1 encodes a non-structural protein about 1693 amino acids (aa) long, with at least four putative functional domains: methyltransferase, cysteine protease, helicase and RNA-dependent RNA polymerase (RdRp). Other domains homologous with those of other plant and animal positive-stranded RNA viruses have been described: the Y domain, the polyproline region (PPR), also called the hypervariable region (HVR), and a macro domain, also called the X domain [2]. Sequence analyses suggest that the Y domain is an integral part of the methyltransferase [3]. It is still unclear whether the ORF1 polyprotein is processed into individual proteins [2]. HEV1 was recently shown to have an additional reading frame, ORF4, which overlaps with ORF1. Stress on the endoplasmic reticulum induces synthesis of the ORF4 protein, which is required for the proper functioning of HEV RNA polymerase [4]. ORF2 and ORF3 also overlap, and their corresponding proteins are translated from a bicistronic subgenomic RNA. ORF2 encodes the 660 aa capsid protein, which has been divided into three domains: S (shell), M (middle) and P (protruding). It was recently shown that ORF2 also encodes a secreted free form of the capsid protein (ORF2s) that differs from the actual capsid protein, ORF2i (for infectious). ORF2i translation is initiated at a previously unrecognized internal start codon [5]. ORF2s is secreted into the extracellular space as an O, N glycosylated and sialylated dimer. Lastly, ORF3 encodes a 113 or 114 aa phosphoprotein, depending on the genotype. This protein has ion channel activity and is involved in virus egress from infected cells [6]. The HEV circulating in the blood is quasi-enveloped, as it is cloaked in host cell membranes, but it is shed into the feces as un-enveloped virions because lipids have been removed by the action of bile salts [7,8].

**Figure 2:**
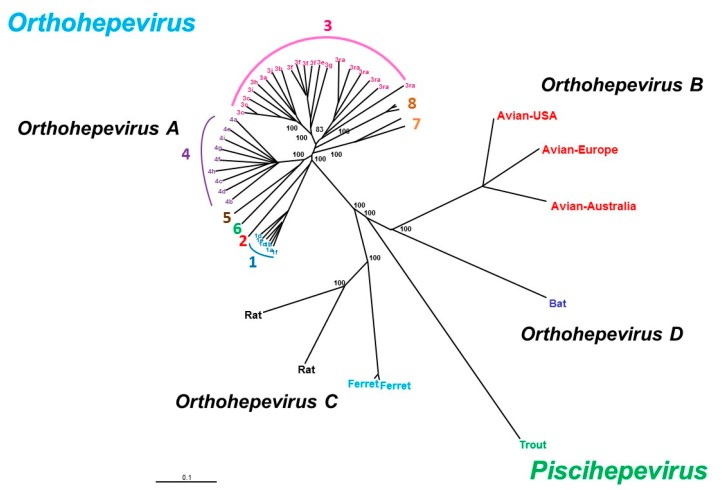
Phylogenetic tree based on full-length sequences of HEV strains. Sequence alignment was performed using ClustalW (MEGA5) and BioEdit, version 7.0. The neighbor-joining method was used to create the phylogenetic tree, with a bootstrap of 100 replicates.

HEV belongs to the *Hepeviridae* family, which has two genera: *Piscihepevirus* (cutthroat trout virus) and *Orthohepevirus* (mammalian and avian strains) with four species (A–D) (Figure 2). The *Orthohepevirus A* HEV species infect humans and other mammals, *Orthohepevirus B* infects chickens, *Orthohepevirus C* infects rats and ferrets, *Orthohepevirus D* infects bats and *Piscihepevirus A* infects the cutthroat trout. The largest species, *Orthohepevirus A,* consists of at least eight distinct HEV genotypes that infect human (HEV1, 2, 3, 4 and 7), pigs (HEV3 and 4), wild boar (HEV3, 4, 5 and 6), rabbits (HEV3), mongooses (HEV3), deer (HEV3), yaks (HEV4) and camels (HEV7 and HEV8) [9]. Only one serotype has been described. While several subgenotypes are described, no consistent criteria have yet been defined to discriminate between virus subgenotypes. HEV3 variants are arranged in three major clades: HEV3abjkchi, HEV3efg, and HEV-3rabbit (ra) based on phylogenetic groupings [10,11]. Two of the four major genotypes, HEV1 and HEV2, only infect humans and are found in developing countries. HEV3 is widely distributed around the world and HEV4 is found mainly in Asia. The HEV3 and HEV4 genotypes are transmitted zoonotically from pigs, wild boar, deer and mongooses [12]. Rabbit strains that are close to HEV3 have been identified in humans [13,14,15]. Camel HEV7 has been described in a liver transplant recipient who had consumed camel meat and milk [16]. HEV5 and HEV6 have been described in wild boar in Japan but not yet in humans. However, Cynomolgus monkeys have been experimentally infected with an HEV5 strain [17]. Cynomolgus macaques are also susceptible to HEV8 [18].

Two cases of patients infected with *Orthohepevirus C* HEV were reported recently, despite their genetic differences from other human pathogenic strains [19,20]. There is, as yet, no evidence that HEV strains can be transmitted from ferrets, bats, birds or trout to humans.

## 3. Clinical Course of HEV Infection

While most HEV infections are asymptomatic, any illness that is caused is usually self-limiting and lasts just a few weeks in the majority of patients. Acute icteric hepatitis, the classic presentation of hepatitis E, occurs in 5%–30% of infected patients. The prodromal phase lasts up to one week and its non-specific symptoms include fever and nausea, vomiting, anorexia or malaise. Dark urine and jaundice mark the onset of the icteric phase. Symptoms usually resolve spontaneously after a few days to a week, but mortality rates can vary from 0.5% to 4.0% of infections during an outbreak [21].

HEV1 and HEV2 mainly infect young adult males (15–30 years) in developing countries and can be asymptomatic, cause mild systemic illness, or icteric acute hepatitis that can be fulminant or lead to acute liver failure. Pregnant women are particularly at risk and a large proportion of those in their second and third trimester of pregnancy can progress to acute liver failure. The mortality rate may reach 25% during the third trimester [22]. Pregnant women die of obstetric complications such as hemorrhage or eclampsia. Fulminant liver failure were also described. Stillbirths are common, as is vertical transmission to infants, which leads to increased neonatal morbidity and mortality [23]. One Indian study found that mortality rates of HEV-related and non-HEV-related acute liver failure in pregnant women were similar, although HEV-related acute liver failure was more common during pregnancy [24]. HEV1 infection during pregnancy is also associated with more frequent miscarriages, preterm deliveries, stillbirths and perinatal mortality.

In developed countries, patients infected with HEV are usually middle-aged or elderly men (>55 years). Severe HEV infections were not described in pregnant women. Patients with underlying liver disease have a poor prognosis in both developing and developed countries [25,26]. In Europe, 5%–33% of patients infected with HEV3 or HEV4 develop symptoms, including jaundice [27,28,29]. The occurrence of symptoms could be linked to the virus load, as recently suggested by a study showing that symptomatic patients had a higher HEV RNA concentration than did HEV RNA-positive healthy blood donors [30].

HEV3 and HEV4 can persist in immunocompromised patients, including solid organ transplant (SOT) patients [31,32,33] and those co-infected with the human immunodeficiency virus (HIV) with a T CD4+ count < 200/mm^3^ [34,35,36]. There have also been reports of chronic HEV infections in patients with hematological disease receiving chemotherapy [37,38,39,40], those given stem cell transplants [41] or patients with rheumatic disorders on heavy immunosuppression immunotherapy [42,43]. Only one case of a chronic HEV1 infection has been reported to date [44], but the finding has been debated because the HEV RNA was not quantified [45]. A patient who had consumed camel meat and milk is also believed to have developed a chronic HEV7 infection [16]. Chronic HEV infection is defined as HEV replication that persists for more than 3 months [46]. A chronic HEV infection can lead to chronic hepatitis and progress rapidly to cirrhosis in 10% of the chronically infected patients [31,32,47,48]. Some of these patients may die from decompensated cirrhosis 2–3 years after the diagnosis. There have been no reports of HEV-infected transplant recipients developing fulminant hepatitis.

## 4. Extra-Hepatic Manifestations

Extra-hepatic manifestations can occur both in patients with acute or chronic HEV infections. They include a range of neurological symptoms and impaired kidney function associated with cryoglobulinemia. Little is known about the mechanisms leading to these extra-hepatic manifestations, although direct viral effects due to HEV replication in affected tissues and indirect effects mediated by the immune system could be involved.

### 4.1. Neurological Manifestations

Neurological disorders have frequently been reported in patients with acute or chronic HEV infections and approximately 150 cases of neurological injury in HEV1-infected Asians and HEV3-infected Europeans have been described to date [49]. These include neuralgic amyotrophy (NA), Guillain–Barré syndrome, Bell’s palsy, and polyradiculopathy [50]. The several cohorts and case-studies of HEV infection in patients with NA have almost all been done on HEV3-infected Europeans. A European study covering four centers in France, UK and the Netherlands prospectively tested over 450 consecutive patients with acute-onset non-traumatic neurological injury. It found that 2.4% of them showed evidence of an HEV infection [50]. The three cases of NA were all HEV-associated. Similarly, an Anglo/Dutch cohort study found that 5/47 (10.6%) patients with NA had evidence of an HEV infection at the onset of their illness [51]. The multi-centric European study of 118 patients with NA showed that patients with HEV-associated disease have a distinct clinical phenotype, compared to patients with NA, without evidence of HEV infection. Patients with HEV-associated NA were significantly more likely to have bilateral involvement of, and more extensive damage to, the brachial plexus. Neurological damage outside the brachial plexus, particularly phrenic nerve involvement, was also more likely in these patients [52]. Lastly, a recent report described finding HEV RNA and intrathecal synthesis of anti-HEV immunoglobulin M (IgM) in a patient with NA, indicating a neurotropic HEV infection [53].

The association between HEV infection and Guillain–Barré syndrome is supported by three case–control studies from Bangladesh [54], Japan [55] and the Netherlands [56]. They found evidence of a recent HEV infection in 5%–11% of patients with Guillain–Barré syndrome, which was a significantly greater frequency than in the healthy control group. Lastly, 6/73 (8%) Belgian patients with Guillain–Barré syndrome had an HEV infection [57].

Other manifestations, like neuropathic pain, painless sensory disorders, encephalitis/myelitis, mononeuritis multiplex, vestibular neuritis, myositis or peripheral neuropathy, have also been described in HEV-infected patients [49,58]. Almost all of these patients with neurological manifestations had normal or modestly abnormal liver function, indicating that the neurological symptoms and signs dominate the clinical picture in these patients.

The pathophysiology of HEV-associated neurological injury remains uncertain, although the immune response triggered by the virus may play a role. A recent study showed that neurological injuries were more frequent in immunocompetent patients (22.6%) than in immunocompromised ones (3.2%, *p* < 0.001) [58], suggesting that Guillain–Barré syndrome and NA are immune-mediated, due to molecular mimicry. This hypothesis fits well with the current thinking about Guillain–Barré syndrome pathophysiology and NA. Another possibility is direct virus neurotropism: pyramidal syndrome was found in a chronically HEV-infected kidney recipient. The variants characterized in the cerebrospinal fluid of this patient differed from those in the serum at the same time, suggesting the emergence of neurotropic variants [59], and possibly HEV replication in the central nervous system.

Using a gerbil model infected intraperitoneally with a 6.57 × 10^7^ genome equivalent of an HEV4 strain isolated from a swine liver sample, negative strands of HEV RNA and ORF2 protein were detected by polymerase chain reaction (PCR) and immunohistochemical staining respectively, in the brains and spinal cords of the animals, suggesting that HEV4 damages the blood–brain barrier and replicates in these tissues. Pathological changes, including neuron degeneration and necrosis, microglia nodules, Purkinje cells necrosis and infiltration by inflammatory cells, were reported [60]. These features parallel the neurological manifestations observed in humans. Immunohistochemical studies on rabbits inoculated with 6.63 × 10^8^ copies via intraperitoneal injection also found the ORF2 antigen in the brains and spinal cords of these animals [61]. Intravenous inoculations of the HEV4 KM01 strain isolated from stools of a swine in mouse 1 × 10^5^ copies/mL and in rhesus macaques (2 × 10^4^ copies) lead to the detection of the ORF2 protein by immunohistochemistry analysis in the cerebellum of the mouse brain tissue and in the granule layer of cerebellum of the rhesus macaque brain [62]. HEV3 Kernow-C1 p6 clone can directly infect human neural cells, neuroblastoma SH-SH5Y, neuroepithelioma SK-N-MC and glioblastoma U87 and U343 cells in vitro [62]. Another study demonstrated that M03.13 oligodendrocytic cells are invaded and support the lifecycle of the HEV3 Kernow-C1 p6 clone when a monolayer of 5 × 10^5^ cells were inoculated with 3 × 10^7^ RNA copies [63]. Human mesodermal and neuroprogenitor cells derived from pluripotent stem cells support HEV replication only when transfected with subgenomic replicon derived from HEV3 Kernow-C1 p6 clone [64]. Lastly, a recent in vitro study showed changes in the tight junction proteins (including Claudin 5, Occludin and Zonula occludens-1) of primary cultures of human brain microvascular cells (HBMVCs) inoculated with swine HEV4 strain for 48 h at a multiplicity of infection of 300 [61]. This observation could explain how HEV crosses the blood–brain barrier to access the central nervous system. However, another group fails to show susceptibility of mice to HEV1, HEV3 or HEV4 [65]. This could be due to the difference of viral isolates used for the different experiments. Whether only specific HEV strains are able to infect brain tissue remains to be determined.

### 4.2. Renal Manifestations

Both acute and chronic HEV infections can lead to kidney injuries and impaired renal function [66,67], but little is known about the underlying mechanisms. Renal biopsies from patients infected with HEV1 or HEV3 show signs of glomerular disease [67], including membranoproliferative glomerulonephritis (MPGN) with or without cryoglobulinemia and membranous glomerulonephritis [67,68,69]. HEV may also trigger a flare-up of a pre-existing IgA nephropathy [67,70]. HEV RNA has been detected in the cryoprecipitate from the serum of an immunocompetent patient with an acute HEV infection who presented with cryoglobulinemic MPGN [69], and HEV infection was found to be an independent predictive factor for cryoglobulinemia in SOT recipients [71]. Renal function improves after HEV clearance, and proteinuria is reduced in most patients [67,69,70].

Patients with a hepatitis C virus (HCV) infection and kidney MPGN disease can develop deposits of immune complexes formed from the HCV antigen, anti-HCV IgG antibodies and a rheumatoid factor in their glomeruli [72]. Perhaps a similar mechanism is at work in HEV infections. Both HEV antigen and RNA have been detected in the urine of patients with chronic HEV4 [73] or HEV3 infections [74]. High concentrations of HEV antigen were found in the urine of immunocompromised patients, independently of the detection of HEV RNA. High molecular weight ORF2 molecules, like HEV virions, should not freely cross the glomerular filtration barrier [5,75,76], but lower molecular weight by-products of ORF2s could be secreted into the urine and be detected by the assay used. It is also possible that the HEV antigen could be secreted into the urine by kidney epithelial cells. However, there is still no evidence that HEV is directly nephrotoxic or that it can replicate in human renal cells. HEV-positive and negative strand RNA and ORF2 protein have also been detected in the kidneys of infected animals, especially swine [77], gerbils [78], monkeys [73] and rabbits [79]. Histopathological analyses of kidney biopsies identified tubule-interstitial with interstitial inflammatory cell infiltrates [73,78,79]. Immunohistochemistry also detected ORF3 protein in the kidneys of infected rabbits [80]. This suggests that the kidneys or the urinary tract could be an HEV reservoir, although little is known of the pathogenicity of HEV for the human kidney.

HEV can also have hematological manifestations such as anemia and severe thrombocytopenia. Hemolytic anemia was reported in HEV-infected patients with a glucose 6-phosphate dehydrogenase (G6PD) [81,82,83,84]. A Pakistani male patient developed aplastic anemia following severe acute hepatitis E [85], while Woolson et al. reported that 12/106 (11%) HEV-infected patients had low platelet counts [86]. Just how HEV induces thrombocytopenia is unknown. It could be immune-mediated as in other virus infections or be linked to the development of fibrosis with splenomegaly. Lastly, several cases of HEV-induced acute pancreatitis have been reported [87,88,89,90,91]. A single-center study from India found that 2.1% (16/790) of patients with acute pancreatitis had serological evidence of a recent HEV infection with no other discernible cause of pancreatitis [92]. The authors suggested that this was due to edema developing in the ambulla of Vater, which then obstructs pancreatic fluid flow.

## 5. Pathogenesis

The pathogenesis of hepatitis E remains poorly understood. It is unclear how, and in what form, the virus particles reach the liver since it is transmitted by the fecal oral route. Recent data showed that primary cultures of intestinal cells support HEV1 and HEV3 replication, and HEV RNA and ORF2 antigen have been detected in the intestinal crypts of a chronically infected patient [93]. These elements suggest that HEV first replicates in the intestinal tract before reaching the liver via the blood in a quasi-enveloped form. It could then replicate in the cytoplasm of hepatocytes and be released as lipid-associated particles into the blood and bile. Capelli et al. used polarized hepatocytes in vitro to show that most HEV particles are released at the apical membrane, that is, the bile side [94]. Bile salts then strip the lipids from the virus shed in the stool. Since HEV is not cytopathic, the liver damage induced by an HEV infection may be immune-mediated by cytotoxic T cells and natural killer cells [95].

### 5.1. Innate Immune Response

The innate immune response during an acute or chronic HEV infection is poorly understood and has not been studied intensively. Microarray analyses of the intrahepatic transcriptome in serial liver biopsies obtained from chimpanzees infected with HEV1 or HCV suggest that the innate immunity mediated by interferon (IFN)-α restricts the replication of HEV more efficiently than that of HCV [96]. Comparison of the results of this study with those for hepatitis A virus (HAV)-infected chimpanzees [97] indicates that HEV triggers a stronger IFN response than does HAV. Analysis of the rhesus macaque liver gene expression showed that the profile differs depending on the genotype (HEV1 or HEV3) of the strain used for infection [98]. HEV1 and HEV3 infections may trigger different host mechanisms to control viral infection: 25% of the interferon-responsive genes were down-regulated during early viremia following an HEV1 infection, including interferon regulatory factor (IRF)3 and IRF7, or interferon-stimulated gene (ISG)15. These same genes were up-regulated during HEV3 infection. The origin of the cytokines, especially type 1 IFN, remains to be determined, since liver biopsies contain both infiltrating leukocytes and infected hepatocytes.

**Figure 3:**
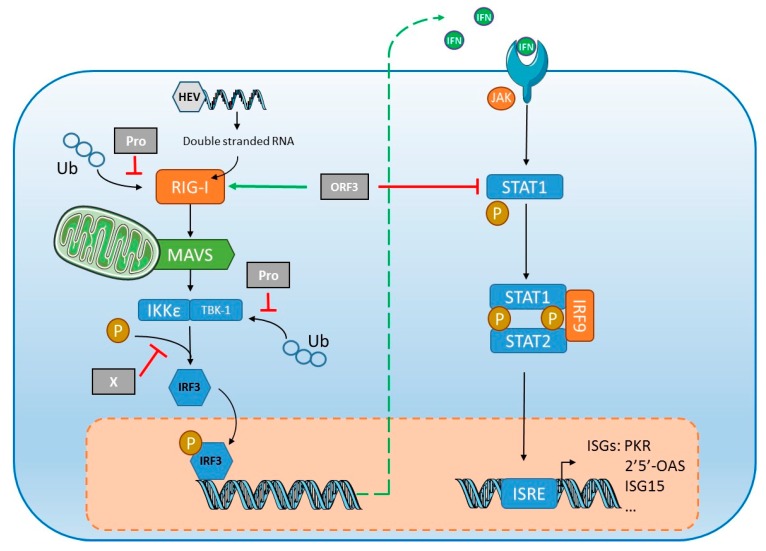
Interference of HEV with the innate antiviral response. HEV RNA is detected in the cytoplasm by the retinoic acid-inducible gene I (RIG-I), leading to type I and type III interferon (IFN) production. The protease domain (Pro) of the ORF1 protein inhibits signaling via RIG-I and prevents IFN induction by de-ubiquitining RIG-I and TANK binding kinase 1 (TBK-1). The X domain (X) inhibits the phosphorylation (P) of IFN regulatory protein 3 (IRF3). Conversely, the ORF3 protein stimulates the direct interaction of type I IFN with RIG-I, but ORF3 also binds to STAT1 to restrict its phosphorylation and activation of the downstream cascade, thus inhibiting the expression of the IFN-stimulated genes (ISGs), including the double stranded (ds)RNA-activated protein kinase (PKR) and 2′,5′-oligoadenylate synthetase (2′5′-OAS) or ISG15. IKKε: IκB-Kinase-epsilon; IRF9: IFN regulatory protein 9; ISRE: interferon stimulated response element; MAVS: mitochondrial antiviral-signaling protein; Ub: ubiquitin.

However, HEV has developed mechanisms to suppress IFN-α signaling (Figure 3). In vitro studies on human lung epithelial A549 cells [99] and hepatocarcinoma Huh7 cells [100] indicate that interferon-induced phosphorylation of the signal transducer and activator of transcription STAT1 are inhibited by the ORF3 protein, blocking the synthesis of two key antiviral proteins, double stranded (ds) RNA-activated protein kinase (PKR) and 2′,5′-oligoadenylate synthetase (2′5′-OAS) (Figure 3). A group showed that ORF3 protein enhanced type I IFN production by HEK293T cells by interacting directly with the pattern recognition receptor (PRR) retinoic acid-inducible gene I (RIG-I) [101]. This group used the same in vitro system to show that ORF1 protein inhibits RIG-I signaling and prevents IFN-β induction by de-ubiquitination of RIG-I and tank binding kinase 1 (TBK1) [102]. The inhibitory effect is rather small, contributing in a minor way to HEV’s resistance to IFN, but silencing the key component gene of the Janus Kinase (JAK)-STAT cascade of IFN signaling, including JAK1, STAT1 and IRF9, stimulates HEV infection and replication, indicating that the IFN cascade can restrict HEV infection [100]. Analyses of gene/protein expression in A549 cells infected with HEV showed the robust induction of inflammatory cytokines/chemokines including TNF-α, IL-6, IL-8 and RANTES (regulated on activation, normal T cell expressed and secreted). HEV infection also led to activation of both NF-κB and IRF3, two transcription factors activated in innate immune signaling pathways [103]. These results obtained with different cell lines need further confirmation in other systems, like primary hepatocytes and/or in vivo animal models. Using primary human hepatocytes, it was shown that HEV was able to persist despite production of type III IFNs [104], suggesting that HEV may more efficiently block the signaling pathway of IFNs rather than their production in a more physiological system. Elevated ISG expression was detected in the livers of chimeric mice engrafted with human hepatocytes without adaptive immunity [105]. Conversely, another study found no intrahepatic ISG induction in humanized uPA/NOG mice infected with HEV1 or HEV3 [106], but the earliest tests were done two weeks post-inoculation, which would have missed an early, transient ISG induction. Lastly, high concentrations of IFN-λ3 were found in the serum of patients at the early phase of acute HEV infection [107]. In vitro experiments with A549 or human epithelial colorectal adenocarcinoma Caco-2 cells also showed that IFN-λ3 inhibited HEV replication in a dose-dependent manner [107]. These results need further confirmation using hepatocarcinoma cell lines like HepG2. Interestingly, even though HEV can replicate in the presence of IFNs, HEV can be cleared when infected HepG2 cells were treated with high doses of IFNs for a prolonged period [104].

Natural killer (NK) and natural killer T (NKT) cells constitute a major fraction of the lymphocytes in the liver, where they are important for the pathogenesis of viral hepatitis. Consequently, these cells could also play a major role together with infected hepatocytes in the innate immune response to HEV. The peripheral blood of acutely infected patients contains a higher proportion of CD4+ cells than in the blood of uninfected controls, but that of CD8+ cells is unchanged [108]. However, this increase in CD4+ cells is not associated with an expansion of HEV ORF2-specific CD4+ CD69+ cells producing helper T cell type 1 (IFN-*γ* and TNF-*α*) cytokines or helper T cell type 2 (IL-4) cytokines. The authors suggest that the expansion of CD4+ cells could reflect an increase in NKT cells, which can be either CD3+ CD4+ or CD3+ CD4− CD8− [108]. Another study showed that there are significantly fewer NK (CD3− CD56+) and NKT (CD3+ CD56+) cells in peripheral blood mononuclear cells (PBMCs) during acute hepatitis E than in uninfected controls [109]. However, the blood of patients with acute hepatitis E has a much greater proportion of activated NK cells than does the blood of uninfected controls. The apparent depletion of total NK and NKT cell fractions in PBMCs could reflect the increased migration of these cells to the liver of infected patients or their apoptosis following activation [109]. An immune-histological analysis of liver biopsies from HEV-infected acute liver failure patients showed that their cell counts of CD56+ were significantly higher than in biopsies from patients infected with HAV, HBV or HCV [110]. The number and the degree of activation of mast cells in Mongolian gerbils experimentally infected with HEV4 was also increased in both the liver and small intestine, suggesting that they are implicated in infection [111].

### 5.2. Humoral and Cell-Mediated Immune Responses

**Figure 4:**
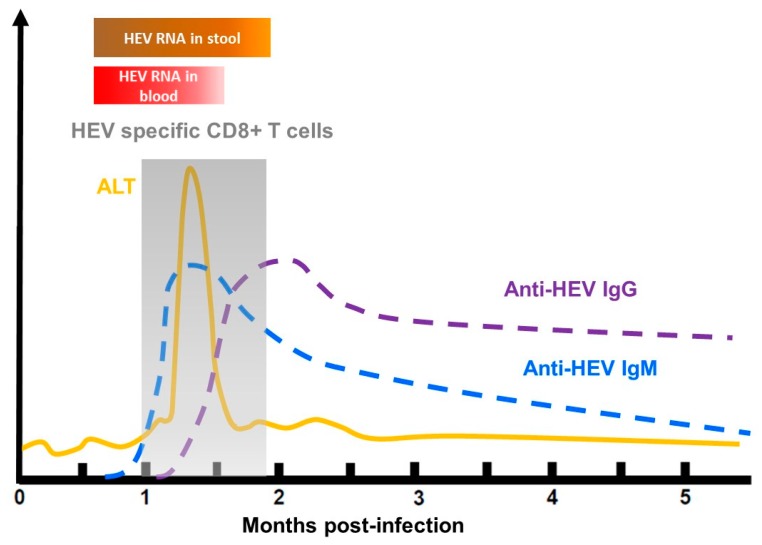
Course of an acute HEV infection. ALT: alanine aminotransferase activity.

HEV-infected patients generally produce a serological anti-HEV response at the onset of illness. Anti-HEV IgMs are present in the early phase of clinical illness, and they can persist for several months. IgM are usually used to diagnose an HEV infection [112]. Anti-HEV IgG appears shortly after the IgM response and can last up to 14 years [113] (Figure 4). The capsid protein contains several neutralizing epitopes and is thus the main target of anti-HEV neutralizing antibodies [114]. Vaccination can also induce anti-HEV antibodies. Hecolin^®^, the only licensed vaccine, is composed of a truncated HEV capsid protein, p239, and confers protection against hepatitis E for up to 4.5 years [115,116]. To date, it is only available in China.

The risk of HEV reinfection is unclear. Cross-protection is possible as there is only one serotype [117]. Studies on non-human primates indicate that anti-HEV antibodies induced by passive or active immunization protect animals against infection. Antibodies also protect humans during outbreaks and non-outbreaks [118,119,120]. Although the minimum protective concentration of antibodies remains to be determined, an antibody concentration of 2.5 World Health Organization (WHO) units/mL was found to be protective in a vaccine study [116,121]. SOT recipients can be re-infected when the antibody concentration is below 7 WHO units/mL [122]. Lastly, the role of ORF2 during natural HEV infection remains to be determined. In vitro studies indicate that ORF2 is not essential for the HEV life cycle, but it reduces antibody-mediated neutralization [5]. Therefore, ORF2s may favor evasion of humoral immunity during HEV infection. This is intriguing since HEV particles in the blood are insensitive to neutralizing antibodies because of their associated lipid [123].

Studies have shown that effector T cells are activated during acute hepatitis E, with more CD8+ cells in infected patients than uninfected controls [124,125]. Husain et al. used an enzyme-linked immunospot (ELISPOT) assay to show that the proportions of PBMCs producing IFN-γ in response to stimulation with ORF2/ORF3 peptides are higher in infected patients than in uninfected controls [124], while the immunohistochemistry studies of Prabhu on liver biopsies from patients with HEV-induced acute liver failure indicate that their liver were infiltrated with activated CD8+ T cells containing granzymes [110].

An increased expression of CD11a integrin in naïve CD45RA+ T cells, as well as overexpression of CCR5 and CCR9, two chemokine receptors that play important roles in cell trafficking and homing, was also reported in peripheral blood of acutely infected patients. The expanded CD45RA+ CD11a high subpopulations present during the early phase of acute infection suggests the recruitment of these cells from the periphery to the liver, thus contributing to the pathogenesis of the infection [125]. Patients with acute HEV infection have a higher proportion of CD4+ CD25+ forkhead box P3+ (FoxP3+) and CD4+ CD25− FoxP3+ regulatory T cells and a rise in IL-10, a signature cytokine of regulatory T cells, than uninfected controls [126]. This suggests that the immunosuppressive immune response is involved in the acute phase of the infection, but its exact role remains to be clarified.

Several studies have reported a broad HEV-specific T cell response to both non-structural and structural proteins [127,128]. HEV-specific T cells are also present in the blood of convalescent patients [127,128] but their frequencies decline rapidly after resolution of the infection [127]. Lastly, specific T cell immunity also seems to confer cross-protection against HEV1 and HEV3 [129], which could protect patients previously exposed to HEV3 when travelling to areas where HEV1 is endemic.

## 6. Pathogenesis of Severe Hepatitis

Fulminant hepatitis is more frequent in men and women suffering from chronic liver disease [25,26,130] and in pregnant women [22].

### 6.1. Severe Hepatitis E in the General Population

The reasons why a HEV infection becomes severe or fulminant are still obscure. It was reported that stimulating both type Th1 and Th2 immune responses could play a role in liver failure. Patients with fulminant hepatic failure (FHF) have higher anti-HEV IgM and IgG concentrations than those with self-limiting infections [131]. Similarly, PBMCs from patients with FHF produce higher IFN-γ, TNF-α, IL-2 and IL-10 concentrations after stimulation with ORF2 peptides than do PBMCs from healthy controls [131]. In contrast, another study reported that the antiviral cellular immune responses and heightened humoral antiviral responses in patients with fulminant hepatitis E were less marked than those of patients with uncomplicated infection or healthy controls [132]. The heightened humoral response was associated with a more severe HEV disease in both studies. The situation in peripheral blood may not reflect that at the site of infection. CD4+ T cells are more frequent in the livers of patients with FHF due to HEV [110] and CD8+ T cells have been shown to infiltrate the livers of patients with fulminant hepatitis E [110,133]. Thus, cytotoxic CD8+ T cells could be particularly important in the pathogenesis of fulminant hepatitis. It was shown, recently, that liver damage during an HAV infection is mediated by the innate-like cytotoxicity of IL-15-activated bystander CD8+ T cells, i.e., stimulation of unrelated (heterologous) T cells by cytokines during an antigen-specific T-cell response [134]. Whether the liver damage is mediated in this way during HEV infection remains to be determined.

Viral factors may also be important. For example, HEV4 infections tend to be more severe than those due to other genotypes [135]. A Belgian study also found that the risk of HEV3c-infected patients being hospitalized was lower than that of patients infected with HEV3f [136]. The severity of an infection could be linked to the genotype and/or the subgenotype, perhaps due to specific mutations in the polyprotein. The HEV1 strains from six Indian patients with FHF contained six mutations in the ORF1 polyprotein (F179S, A317T, T735I, L1110F, V1120I and FG1439Y) which were not found in the strains from patients with uncomplicated acute hepatitis E [137]. However, Smith et al. re-examined the association between FHF and HEV genotypes and concluded that host factors are responsible for the development of fulminant hepatitis rather than virus genotype, variants or specific aa substitutions [138]. Despite this analysis, one recent study found two mutations (C1483W and N1530T) in the HEV1 polymerases from all 25 patients with acute liver failure but in none of the patients with acute hepatitis E [139]. The same group also identified three mutations in the methyltransferase (V27A, D29N, H105R) of the HEV1 from 16 patients with acute liver failure, which were not detected in patients with uncomplicated acute hepatitis E [140]. Consequently, the real impact of virus polymorphism on disease severity still needs to be clarified.

### 6.2. Severe Hepatitis in Pregnant Women

The cause of elevated maternal mortality (30%, with most deaths occurring in the third trimester) of pregnant women infected with HEV1 living in developing countries has been the subject of many studies. HEV genotype could explain the poorer outcome in pregnant women, at least in part, since HEV3 is not particularly deadly for pregnant women [141,142,143]. There are no data available for HEV4 infection during pregnancy. A recent study demonstrated that HEV1 replicates more efficiently than HEV3 ex vivo in tissue explants of decidua basalis and fetal placenta, and also in stromal cells [144]. HEV1 is associated with increased apoptosis and necrosis at the maternal–fetal interface with alterations in the architecture of the placental barrier. HEV1 also produces more infectious virions and triggers the production of a panel of pro-inflammatory cytokines like IL-6 and chemokines. These changes in the cytokine microenvironment correlate with virus load and contribute to tissue damage [144]. Such findings are consistent with data correlating the increase in several pro-inflammatory factors (TNF-α, IL-6, IFN-γ and TGF-β1) in the peripheral blood of HEV1-infected women with adverse pregnancy outcomes [145].

However, exactly how severe liver injury occurs during pregnancy remains unclear. Pregnancy is associated with changes in the sex hormone profile and the immune system to protect the fetus from the maternal immune system. A shift from a Th1-dominated immune response to a Th2-dominated one, “Th2 bias”, may help protect the fetus by suppressing macrophage activation [146]. Although the existence of a Th2 bias in HEV-infected pregnant women was confirmed, its implication for the severity of the infection is unknown [147]. Women with acute liver failure have a reduced expression of toll-like receptor (TLR) 3/TLR7/TLR9, the key pattern recognition receptor in innate immunity, and their phagocytic macrophages are weaker than those of women with acute viral hepatitis E [148]. However, the phagocytic capacities of monocytes from the two groups are essentially the same. Impaired monocyte-macrophage function in pregnant women with acute liver failure could contribute to an inadequate innate immune response, and hence to the development and severity of acute liver failure.

Other host factors, such as nutritional status, including micronutrient or folate deficiencies, or differences in major histocompatibility complex, may also influence the immune response of pregnant women to HEV [149]. This may be why HEV infections are benign in pregnant women in Egypt, although they are caused by HEV1 [150]. Lastly, pregnancy-associated hormones can also contribute to a poor outcome. HEV-positive pregnant women who develop FHF have higher concentrations of estrogen, progesterone and β-human chorionic gonadotrophin (β-HCG) than HEV-negative pregnant women with FHF or healthy controls [151]. An in vitro study showed that serum from pregnant women, especially those in the third trimester, enhanced the replication of HEV by inhibiting estrogen receptors and the synthesis of type I IFNs [152]. While some studies have found that the high HEV RNA concentrations in HEV-infected pregnant women were associated with a poor outcome [153,154,155], another study has not confirmed these results, with HEV RNA detected only in 1/7 pregnant women [131].

## 7. Pathogenesis of Chronic Infection in Immunocompromised Patients

Most studies on the pathogenesis of chronic HEV installation have focused on SOT recipients. The incidence of HEV infection in these patients varies from 0.9% to 3.5%, based on detecting HEV RNA, and acute infections become chronic in ~60% of them [48]. Other studies that were interested in the seroconversion rate among transplant recipients have reported rates of progression to chronicity ranging from 21% to 50% [156,157,158].

Whether an acute HEV infection resolves without sequelae or progresses to chronic hepatitis seems to be related primarily to the host’s immune response. The ISGs response of renal transplant recipients who did not clear their HEV infection was higher than that of patients who cleared their HEV [159]. This suggests that activation of the IFN signaling pathway does not always lead to spontaneous HEV clearance. The increased expression of ISGs in patients with a chronic HEV infection seems to favor virus persistence by causing the IFN signaling pathway to become refractory [159]. HEV can persist in vitro in human hepatoma cells and primary human hepatocytes despite the continuous production of type III IFNs [104]. In addition, the persistent activation of the JAK/STAT signaling rendered infected cells refractory to exogenous IFN [104]. Low concentrations of IL-1Ra and sIL-2R, together with high concentrations of chemokines, during the acute phase, are also associated with HEV persistence [160]. An HEV infection is likely to become chronic in profoundly immunosuppressed patients. The numbers of CD2+, CD3+ and CD4+ T cells are significantly lower in these patients than in those who spontaneously eliminate the virus [33]. Chronic HEV infections are more frequent in patients who are also infected with HIV, especially those with a low CD4+ T cell count [34,35,36]. In addition, the HEV-specific T cell proliferative responses of SOT patients, especially those with a chronic infection, are weaker. The development of HEV-specific IFN-γ-producing cells seems to be associated with a favorable outcome in SOT patients [161,162]. Another study has shown that the γδ cells of SOT patients are mobilized during the acute phase of infection [163]. Immunocompetent patients do not produce such an immune response, suggesting that SOT patients mobilize a larger fraction of their immunity due to immunosuppressive drug treatment. The antiviral role of these cells at the acute phase of infection needs further investigation.

The immunosuppressive regimen can also influence the development of a chronic infection. Pigs given cyclosporin, azathioprine and prednisolone and infected with HEV3 developed chronic infections [164]. In humans, HEV is more likely to be persistent in SOT patients treated with tacrolimus rather than cyclosporin [48]. Cyclosporin and tacrolimus are both immunosuppressive drugs that inhibit the calcineurin phosphatase in lymphocytes, but tacrolimus impairs the specific T cell response more efficiently than does cyclosporin [165]. In vitro studies have shown that both of these calcineurin inhibitors promote HEV replication by inhibiting cyclophilins A and B, while mycophenolic acid, an inhibitor of inosine 5′ monophosphate dehydrogenase, inhibits HEV replication [166]. Rapamycin and everolimus also promote HEV replication in vitro by inhibiting the mechanistic target of rapamycin (mTOR), showing that the PI3K-PKB-mTOR pathway acts as a cell restriction factor [167]. In line with this observation, patients given mTOR inhibitors have higher HEV RNA concentrations in the blood, while those given mycophenolic acid do not have lower HEV RNA concentrations [168].

Lastly, virus factors may favor the persistence of HEV. Greater quasi-species heterogeneity in the ORF1 and ORF2 regions during the acute phase of infection is associated with HEV persistence [160,169]. Chronic infection was found to be rare in a large cohort of Japanese liver transplant recipients, suggesting that there are differences in HEV subtype, strains, or host genetic factors that influence persistence [170,171]. Fulminant hepatitis has not been reported in HEV-infected transplant recipients.

**Figure 5:**
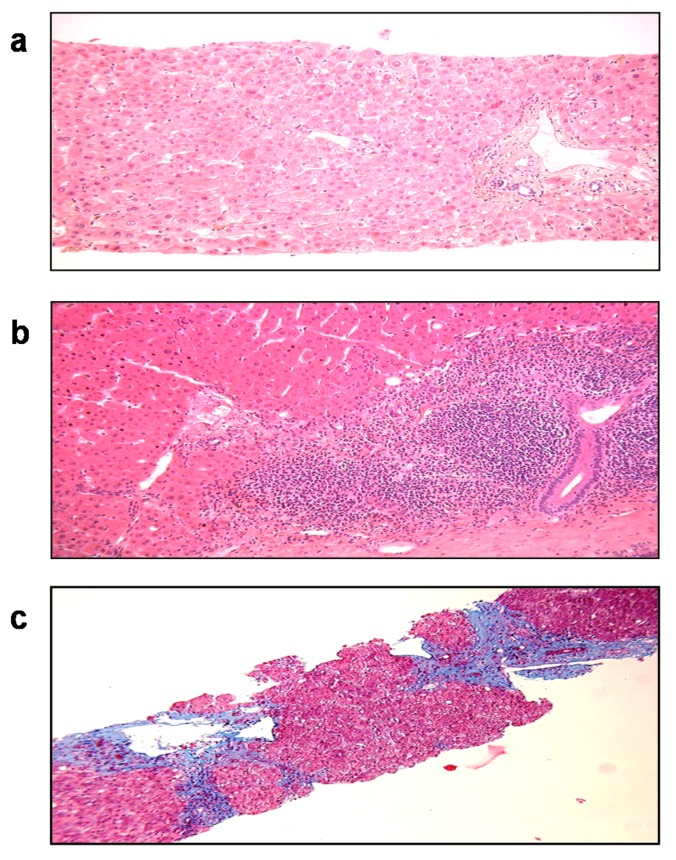
Histology of liver biopsies from a chronically HEV-infected patient. (**a**) Initial liver biopsy, (**b**) inflammation after 15 months of infection, and (**c**) Cirrhosis after 38 months of infection (Masson’s trichrome, magnification 100×).

Nearly 10% of SOT patients with HEV develop cirrhosis within 3 to 5 years after the primary infection (Figure 5). Progression to liver fibrosis seems to be associated with the slow diversification of the P domain of the capsid [160]. More aggressive variants may be selected in fibroses, although this needs confirmation in a larger cohort.

Lastly, chronically infected patients have recently been found to harbor recombinant HEV-host variants [172,173,174]. The PPR regions of these recombinant variants included fragments of human genes of varying origin (inter alpha trypsin inhibitor (ITI-H2), ribosomal genes S17 or S19 and tyrosine aminotransferase). Variants harboring the S17, S19 or ITI fragment had a replicative advantage in vitro, while the impact of TAT was not studied. Duplications and insertions of the HEV genome were also described [173,175]. Their influence on HEV infection is unknown and needs further investigation.

## 8. Treatment

There is no recommended treatment for acute HEV infections. They are usually self-limiting with spontaneous HEV clearance. Ribavirin has been suggested for some cases of immunocompetent patients with severe hepatitis [176], but as there was no control group, it is difficult to claim that ribavirin really improved the course of the infection.

Most studies of the treatment of chronic HEV infection have involved SOT patients. Since the intensity of the immune host response favors HEV persistence, reducing immunosuppressive drugs, particularly those targeting T cells, is the initial option. It leads to HEV clearance in nearly 1/3 of chronically HEV-infected SOT recipients [177]. Antiviral therapy is required for the remaining patients. Pegylated-interferon-α has been successfully used to treat some liver transplant recipients and a hemodialysis patient who cleared HEV after a three-month course of therapy [178,179,180]. However, interferon is generally contraindicated in kidney-, pancreas-, heart- and lung-transplant recipients because it increases the risk of acute rejection by stimulating the immune system.

Ribavirin monotherapy has been extensively studied for treating chronic HEV infections in SOT recipients [158,181,182]. The sustained virological response (SVR) of 59 SOT recipients was 78%, after treatment with ribavirin (median dose: 600 mg/day; range: 29–1200) for three (range, 1–18) months as part of a multicenter retrospective study. Relapse patients who were retreated with ribavirin for a longer period (six months) cleared the virus and achieved SVR, increasing the overall SVR to 90% [183]. The optimal duration of treatment has still to be determined. Treatment should be continued for a further three months if HEV RNA is detected in the stool at the end of the scheduled duration [184]. Thus, HEV RNA in the stools should be monitored before stopping therapy. Ribavirin may block HEV production, as the active metabolite ribavirin monophosphate is a competitive inhibitor of cellular enzyme inosine monophosphate dehydrogenase (IMPDH). In vitro experiments suggest that ribavirin activity results in depletion of the intracellular guanosine triphosphate (GTP) pools, which impedes RNA replication by inhibiting IMPDH [185]. Ribavirin may also act by inducing mutagenesis of the HEV genome [186], therefore disrupting virus replication due to error catastrophe [187]. Mutations in the polymerase, especially the 1634R mutation, have been found in two patients with ribavirin failure [188], but its detection at baseline does not preclude treatment failure [189]. Other mutations in the HEV polymerase have been described: K1383N, D1384G, K1398R, V1479I and Y1587F [186,190], but their presence before treatment does not influence SVR [191].

No other antiviral therapy is presently known to be effective against a chronic HEV infection. Sofosbuvir was shown to have antiviral activity against HEV in vitro [192] but had limited efficacy in HEV-infected patients [193,194,195,196,197,198,199,200]. A recent pilot study of nine patients (seven for whom ribavirin had previously failed) given sofosbuvir (400 mg/day) for 24 weeks found that their HEV viremia decreased, but was not cleared. This suggests that sofosbuvir alone is not a cure for HEV [201]. Zinc salts can block HEV replication in vitro by inhibiting RNA polymerase [202]. However, two female transplant-recipients with chronic HEV infections did not respond to ribavirin despite having elevated intra-erythrocyte zinc concentrations [203]. Clearly, further work with a larger cohort is needed. The natural compound silvestrol blocks HEV replication in vitro [204], and the HEV RNA concentrations in the feces of treated mice rapidly decreased. Whether this compound has a similar action in humans remains to be determined. Screening to identify new anti-HEV drugs found two novel HEV antiviral candidates: NITD008, a broad-spectrum chain-terminating adenosine nucleoside analogue initially developed to treat dengue virus, and GPC-N114, which binds to the RNA channels of picornavirus polymerases [205,206]. These compounds are promising HEV antiviral candidates. Lastly, T cell therapy may be an alternative to drugs [207].

## 9. Conclusions

The pathogenesis of HEV infection involves a complex interplay between the virus and the host immune response. While the clinical phenotype of an HEV infection is primarily hepatological, the range and incidence of HEV-associated clinical symptoms is much broader, including neurological and renal disorders. Ribavirin is currently the treatment of choice for chronically infected patients but there is an urgent need to find other compounds with which to treat patients who fail to clear HEV after ribavirin therapy.
